# Characterization and modulation of anti-αβTCR antibodies and their respective binding sites at the βTCR chain to enrich engineered T cells

**DOI:** 10.1016/j.omtm.2021.06.011

**Published:** 2021-06-24

**Authors:** Guido J.J. Kierkels, Eline van Diest, Patricia Hernández-López, Wouter Scheper, Anja C.M. de Bruin, Elselien Frijlink, Tineke Aarts-Riemens, Sanne F.J. van Dooremalen, Dennis X. Beringer, Rimke Oostvogels, Lovro Kramer, Trudy Straetemans, Wolfgang Uckert, Zsolt Sebestyén, Jürgen Kuball

**Affiliations:** 1 Center for Translational Immunology, University Medical Center Utrecht, Utrecht University, Heidelberglaan 100, 3584 CX Utrecht, the Netherlands; 2 Department of Hematology, University Medical Center Utrecht, Heidelberglaan 100, 3584 CX Utrecht, the Netherlands; 3 Max-Delbrück-Centrum für Molekulare Medizin (MDC), Robert-Rössle-Strasse 10, 13125 Berlin, Germany

**Keywords:** immunotherapy, T cell engineering, select-kill strategy, epitope mapping, T cell depletion, T cell repector (TCR), advanced therapy medicinal products (ATMP), good manufacturing practice (GMP)

## Abstract

T cell engineering strategies offer cures to patients and have entered clinical practice with chimeric antibody-based receptors; αβT cell receptor (αβTCR)-based strategies are, however, lagging behind. To allow a more rapid and successful translation to successful concepts also using αβTCRs for engineering, incorporating a method for the purification of genetically modified T cells, as well as engineered T cell deletion after transfer into patients, could be beneficial. This would allow increased efficacy, reduced potential side effects, and improved safety of newly to-be-tested lead structures. By characterizing the antigen-binding interface of a good manufacturing process (GMP)-grade anti-αβTCR antibody, usually used for depletion of αβT cells from stem cell transplantation products, we developed a strategy that allows for the purification of untouched αβTCR-engineered immune cells by changing 2 amino acids only in the TCRβ chain constant domain of introduced TCR chains. Alternatively, we engineered an antibody that targets an extended mutated interface of 9 amino acids in the TCRβ chain constant domain and provides the opportunity to further develop depletion strategies of engineered immune cells.

## Graphical abstract

**Figure:**
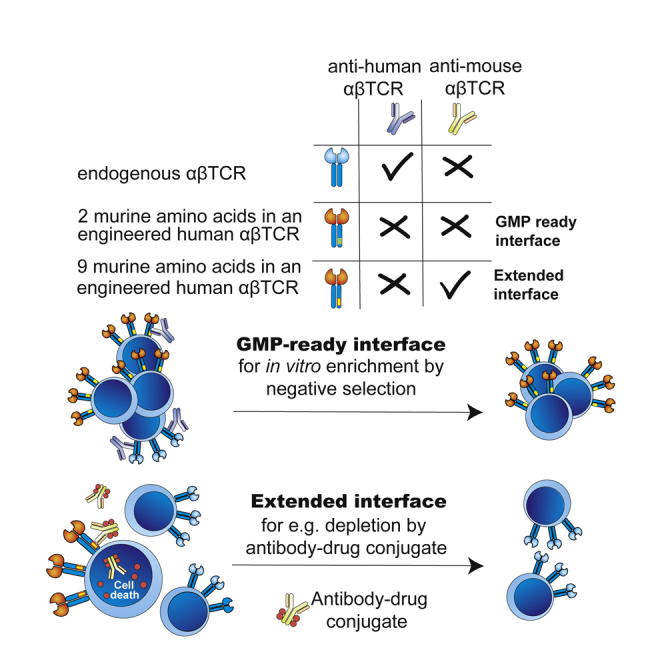

## Teaser

The authors provide a GMP-ready solution for the enrichment of human αβTCR-engineered T cells by murinization of 2 aa. An extended murinized epitope with a total of 9 aa could become a novel interface to specifically target TCR-engineered T cells with an anti-mouse αβTCR antibody.

## Introduction

The US Food and Drug Administration (FDA) approval of the first engineered T cells expressing chimeric antigen receptors (CARs) has paved the way for new cellular interventions in the clinic.^1^^,^^2^ A next wave of cell therapy will come with T cell receptor (TCR)-engineered T cells specific for targets on both solid and hematological malignancies.^3^ Most clinical trials using αβTCR-engineered T cells are directed against cancer/testis antigens, such as NY-ESO-1.^4^ Although the clinical response rates are very encouraging, only a small proportion of the patients benefit from these novel treatments.^5^^,^^6^ Disappointing response rates can be partially attributed to the presence of non-engineered and poorly engineered T cells in the administered cell product.^7^ These non-engineered and poorly engineered T cells can hamper the therapeutic efficiency of engineered immune effector cells because of, e.g., insufficient expression of the introduced receptor, mispairing of introduced αβTCR with endogenous αβTCR,^8^ or by competition for endogenous homeostatic cytokines.^7^^,^^9^ Furthermore, in an allogenic setting, the presence of T cells still expressing the endogenous αβTCR can lead to severe graft-versus-host disease. Purification of engineered T cells before infusion can overcome these hurdles, ultimately resulting in enhanced *in vivo* activity. Current methods for purification of engineered T cells often depend on the expression of artificial molecules such as truncated CD34^10^ or truncated NGFR,^11^ in addition to the tumor-specific receptor. However, larger transgene cassettes used to introduce multiple proteins are relatively difficult to express, and additional transgenes can add immunogenic properties to the engineered cell product.^12^ Besides purification of engineered T cells to increase effectivity, elimination of engineered T cells after adoptive transfer might be needed in case of cytokine release syndrome^13^ or off-target toxicities, e.g., due to peptide mimicry,^5^^,^^14^ expression of the antigen at low levels in healthy tissues,^14^ or mispairing of introduced with endogenous αβTCR chains resulting in unwanted specificities.^8^ A currently explored solution for the elimination of transferred cells is the co-expression of herpes simplex virus thymidine kinase (HSV-TK) along with the transgene of interest,^15^ mainly limited by the immunogenicity and relatively large size of the HSV-TK gene.^15^ An alternative elegant solution is to introduce a myc-tag into the αβTCR sequence itself, followed by *in vivo* depletion through myc-specific antibodies.^16^ However, introducing artificial genes into the αβTCR might alter downstream signaling by modifying, e.g., its glycosylation.^17^ Selection of engineered T cells and subsequent *in vivo* elimination achieved with a single marker, which has previously been described for CD20,^18^ would be favorable, due to the relatively small transgene cassette and therefore better expression. Even better would be a method where the introduced tumor-specific TCR could also be used for both purification and *in vivo* depletion, and thereby would combine all three properties in one gene: tumor specificity, a selection opportunity of cells expressing the transgene at high levels, as well as an *in vivo* depletion option, which allows for the elimination of the engineered immune cells in case of toxicities caused by the introduced receptor. Within this context we have explored a strategy based on the recent development of purified T cells engineered to express a defined γδ T cell receptor (TEGs).^19^, ^20^, ^21^, ^22^, ^23^, ^24^, ^25^, ^26^, ^27^, ^28^, ^29^ In this strategy we took advantage of the observation that an anti-human αβTCR antibody used for the purification of TEGs does not cross-react with γδTCR chains, and it can thereby differentiate between engineered and non-engineered cells. This anti-human αβTCR antibody is routinely used to deplete αβTCR T cells from apheresis products using CliniMACS depletion before allogeneic stem cell transplantation.^3^^,^^28^ In this study, we describe the translation of the TEG purification procedure into a purification procedure for αβTCR-engineered T cells. We also provide the rationale for the additional development of elimination strategies of engineered immune cells by further modulating the binding site to be selectively targeted by a second independent antibody.

## Results

### Anti-human αβTCR binds an epitope on the TCRβ chain of human αβT cells

**Figure 1:**
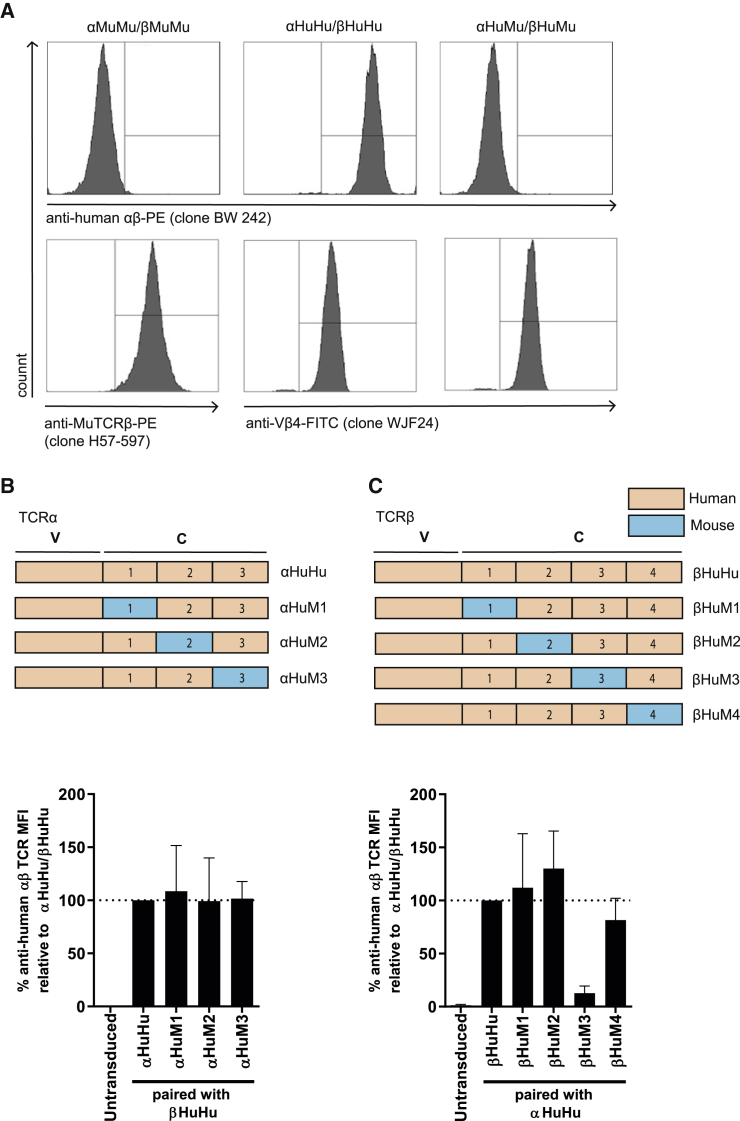
Partial murinization of the TCRβ chain constant domain abrogates binding of the anti-human αβTCR antibody clone BW242/412 (A) Jurma cells were transduced with fully murine (αMuMu/βMuMu), fully human NY-ESO-1-specific (αHuHu/βHuHu) or chimeric αβTCR, in which the α and β constant domains were murine, and the variable domains were human NY-ESO-1 specific. Binding of anti-human αβTCR, anti-MuTCRβ, and Vβ4 was assessed by flow cytometry. (B and C) Schematic representation of the constructed variable (V) and constant (C) domains of αβTCRs that cover all aa differences in the (B) TCRα chain and (C) TCRβ chain (upper panels). The constant domain of the TCRα and β chain have been divided in respectively three or four different regions, based on the comparison of human and murine regions revealing clustered differences flanked by homologous regions as described.^29^ Jurma cells were transduced with the different murinized αβTCRs, after which anti-human αβTCR antibody binding was assessed by flow cytometry. The bar graphs (B and C, lower panels) show the anti-human αβTCR MFI relative to the fully human TCR. Untransduced Jurma cells served as a negative control. The data correspond to two independent experiments, and a representative figure is shown (A) or as the average with standard deviation (B and C).

The good manufacturing practice (GMP)-grade anti-human αβTCR monoclonal antibody clone BW242/412 (from now on referred to as anti-human αβTCR) recognizes a common determinant of the human TCRα/β-CD3 complex, which has not yet been characterized. In order to allow for further epitope mapping of the interface between the anti-human αβTCR clone BW242/412 and a human αβTCR, we first tested the antibody’s ability to bind to murine αβTCRs. Therefore, Jurma T cells, a TCR-deficient T cell line, were transduced with human αβTCRs directed against the cancer/testis antigen NY-ESO-1_157–165_^29^ or with a murine nonsense αβTCR composed of the TCRα chain of an MDM2-specific αβTCR,^30^ and the TCRβ chain of a p53-specific αβTCR.^31^ Specific binding of the anti-human αβTCR was only observed in the human (αHuHu/βHuHu) but not the murine (αMuMu/βMuMu) TCR-transduced Jurma cells (Figure 1A). To rule out that parts of the human variable domain of the αβTCR bind to the anti-human αβTCR antibody, the human NY-ESO-1 αβTCR variable domain was grafted on the murine constant domain to create a chimeric αβTCR (αHuMu/βHuMu). Replacing only the human TCRα and TCRβ constant domains by murine equivalents completely abrogated binding of anti-human αβTCR to levels resembling binding to a fully murine αβTCR (αMuMu/βMuMu). This indicates that the human constant domain contains the binding epitope. Comparable transgenic expression of murine and human TCRs was confirmed by anti-MuTCRβ and anti-Vβ4, respectively (Figure 1A). Infusion of T cells expressing TCRs with complete murine constant domains into patients can generate immunogenic effects and lead to a decreased persistence of the engineered cells *in vivo*.^32^ To minimize these undesirable effects, we aimed to map the minimal amount of murine residues needed to disrupt binding of anti-human αβTCR by making use of previously described chimeric TCRα and β chains, with mutational blocks covering all amino acid (aa) differences between the constant regions of human and mouse αβTCRs.^29^ We tested three NY-ESO-1 TCRα chain variants and four NY-ESO-1 TCRβ chain variants, with each containing one murine domain, flanked by complete human aa sequences. Every TCRα chain was paired with the fully human TCRβ chain (βHuHu) (Figure 1B), and every TCRβ chain was paired with the fully human TCRα chain (αHuHu) (Figure 1C) and introduced into Jurma cells, after which binding of anti-human αβTCR was determined by flow cytometry. Antibody binding was significantly impaired in T cells expressing the αβTCR, which includes murine domain 3 (βHuM3), while none of the other chimeric αβTCRs substantially impaired anti-human αβTCR binding (Figures 1B and 1C). βHuM3 TCR expression was confirmed by staining for anti-Vβ4 and was comparable to αHuHu/βHuHu (Figure S1). These results indicate that domain 3 of the TCRβ chain (βHuM3) dictates the binding of anti-human αβTCR.

### Anti-human αβTCR binding can be abrogated by mutating two residues

**Figure 2:**
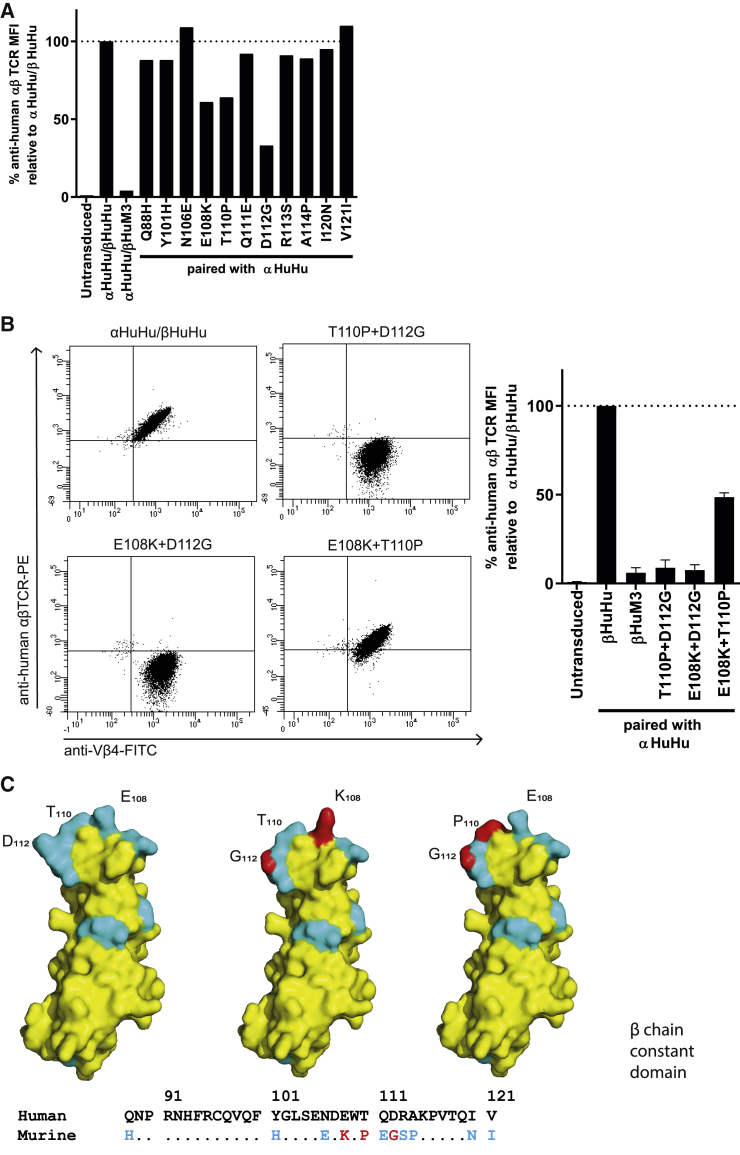
A combination of 2 specific murine aa in the TCRβ chain constant domain is sufficient to abrogate binding of the anti-human αβTCR antibody clone BW242/412 (A) Jurma cells were transduced with αβTCRs containing single murine aa substitutions in the third domain of the β chain, after which binding of the anti-human αβTCR antibody was assessed using flow cytometry. Untransduced Jurma cells served as a negative control, while fully human αβTCR-transduced Jurma cells served as a positive control. (B) Jurma cells were transduced with αβTCRs containing combinations of murine aas in the third domain of the β chain, after which binding of anti-human αβTCR antibody was assessed using flow cytometry. (C) Visualization of the 11 non-homologous aa between human and mouse β chain third domain in cyan using SWISS-MODEL^52^ on the modeled template of the β chain of the human JKF6 T cell receptor (PDB: 4ZDH). Effective single murine aa substitutions are displayed in red. The data correspond to one experiment (A) or two independent experiments shown with a representative image (B) and average with standard deviation (B, bar graph).

Analysis of the sequence of domain 3 of the TCRβ chain constant domain revealed 11 residues that are non-homologous between murine and human species (Figure S2). To determine which residues are essential for anti-human αβTCR binding, we constructed 11 variants of the TCRβ chain, in which each one of the non-homologous aas was replaced by the murine counterpart. These 11 constructs were paired with the completely human αTCR chain (αHuHu), introduced in Jurma cells and tested for binding by the anti-human αβTCR antibody. Of the 11 generated mutants, the substitutions of “human” glutamic acid (E108) to the “murine lysine” (K), “human” threonine (T110) to the “murine” proline (P), and “human” aspartic acid (D112) to the “murine” glycine (G) showed a substantial abrogation of anti-human αβTCR binding (Figure 2A). However, none of these substitutions was sufficient to induce total abrogation, as shown by the TCR consisting of αHuHu/βHuM3 (Figure 2A). Therefore, we constructed TCRβ chains with a combination of the aforementioned mutations. The TCRβ chains with a D112G mutation combined with E108K or T110P were both effective in abrogating binding of the anti-human αβTCR antibody (Figure 2B), which can be explained by a substantial decrease in bulkiness, and thus a decrease in size of these residues (Figure 2C; Table S1). For further engineered T cell experiments, the combination of T110P and D112G murinization was selected.

### Enrichment of αβTCR-engineered T cells utilizing fragments of murine αβTCR chains

**Figure 3:**
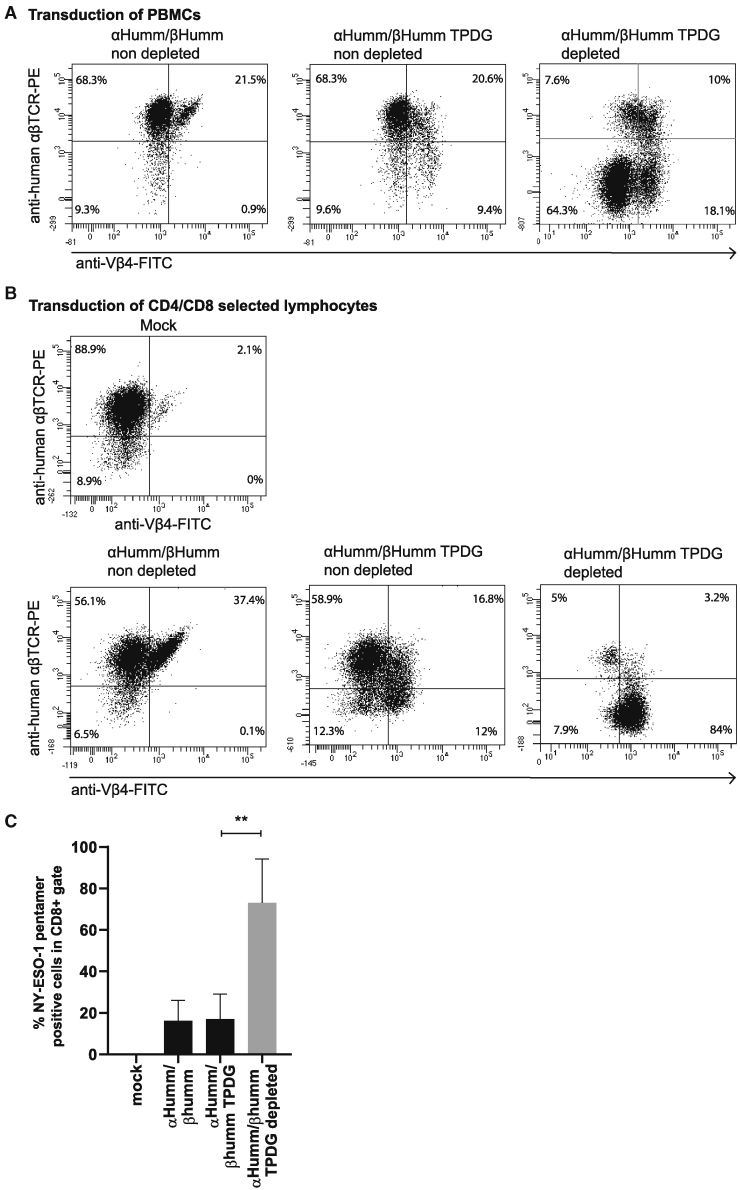
Primary αβT cells engineered with murinized αβTCRs can be successfully selected by using anti-human αβTCR antibody clone BW242/412 to deplete non-engineered and poorly engineered immune cells (A) PBMCs were transduced with minimally murinized αβTCRs with (middle panel) and without (left panel) the “TPDG” mutations. Primary αβT cells with the TPDG mutations were MACS depleted and expanded (right panel). Endogenous αβTCR expression and expression of the introduced αβTCR without the TPDG mutations were determined by flow cytometry using an anti-human αβTCR antibody; expression of the introduced βTCR chain was assessed with an anti-Vβ4 antibody. (B) Prior to transduction with minimally murinized αβTCRs, T cells were selected from PBMCs using CD4/CD8 MACS selection (CD4/CD8^+^). (C) Expression of correctly paired αβTCR chains was assessed before and after depletion and expansion by NY-ESO-1 pentamers (CD8^+^) for both transduction strategies combined. The data correspond to three independent experiments and are shown as a representative figure (A and B) or as average with standard deviation (C). Statistical significance (∗p ≤ 0.05, ∗∗p ≤ 0.01) was calculated using a paired t test.

Murine αβTCRs, or residues derived from murine αβTCRs introduced into human αβTCRs and expressed in human T cells, have been reported to outcompete endogenous human TCR chains.^29^^,^^33^^,^^34^ These murine and murinized αβTCRs preferentially pair with each other, thereby decreasing the occurrence of mispairing with endogenous human αβTCRs. Therefore, we utilized single murine aas to enhance the expression of introduced TCRs.^29^ These “minimally murinized” constant domain variants (from now on referred to as mm) contain murine aas that are both critical and sufficient to improve pairing between the two chains.^29^ Next, we introduced the above-identified murine residues (T110P+D112G) in the TCRβ chain constant domain in order to test whether this was sufficient to disrupt the binding of anti-human αβTCR in human primary T cells. To test this concept, healthy donor T cells were transduced with mm NY-ESO-1-specific αβTCRs as a negative control, or mm NY-ESO-1-specific αβTCRs, including the two identified mutations T110P+D112G. Magnetic-activated cell sorting (MACS) depletion using anti-human αβTCR resulted in an increased cell fraction not able to bind anti-human αβTCR after an expansion of 2 weeks, in order to assess stability of the phenotype (Figure 3A). However, we also observed outgrowth of a large fraction of Vβ4- and αβTCR-negative cells, mainly consisting of natural killer (NK) and γδ T cells, as reported previously.^20^ To further increase purity of engineered immune cells, T cells were selected by CD4/CD8 MACS from peripheral blood mononuclear cells (PBMCs) prior to the transduction. This indeed prevented the outgrowth of NK and γδ T cells after αβTCR depletion and expansion (Figure 3B). Next, we quantified the fraction of NY-ESO-1_157–165_ HLA∗02:01 pentamer^+^ cells before and after depletion, showing a significant increase in pentamer^+^ cells after depletion (Figure 3C), further proving successful enrichment of engineered immune cells when using T110P+D112G-modified αβTCRs.

### Enrichment strategy within the context of alternative αβTCR stabilization procedures

**Figure 4:**
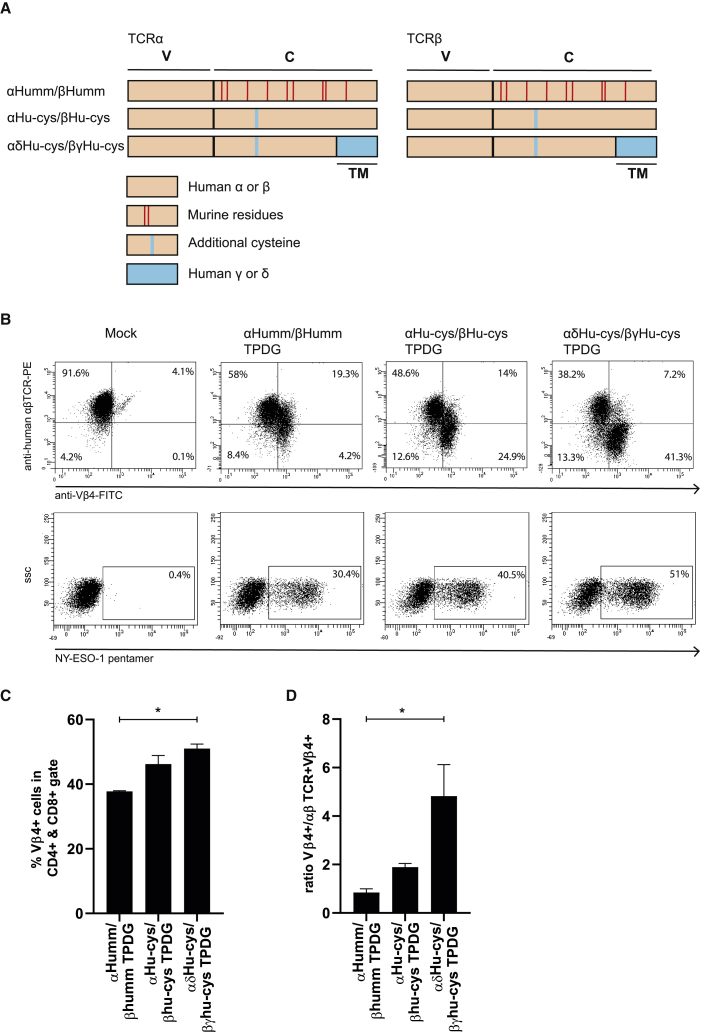
Efficacy of different strategies to induce preferential pairing of introduced αTCR and βTCR chains (A) Schematic representation of the three different methods for creating preferential pairing between the introduced αTCR and βTCR chains. TM, transmembrane domain; V, variable domain; C, constant domain. (B) Primary αβT cells were transduced with the three differentially modified αβTCRs as indicated in (A), and expression of the introduced βTCR was determined by an anti-Vβ4 antibody. (C and D) Pairing of the introduced αTCR and βTCR chains was assessed by NY-ESO-1 pentamers (C), and the percentage Vβ4^+^ cells was quantified for the differently modified αβTCR (D) ratio between Vβ4 single-positive/Vβ4/αβTCR double-positive cells was determined. The data correspond to two independent experiments and are shown as a representative figure (B) or as the average with standard deviation (C and D). Statistical significance (∗p ≤ 0.05, ∗∗p ≤ 0.01) was calculated using a one-way ANOVA.

**Figure 5:**
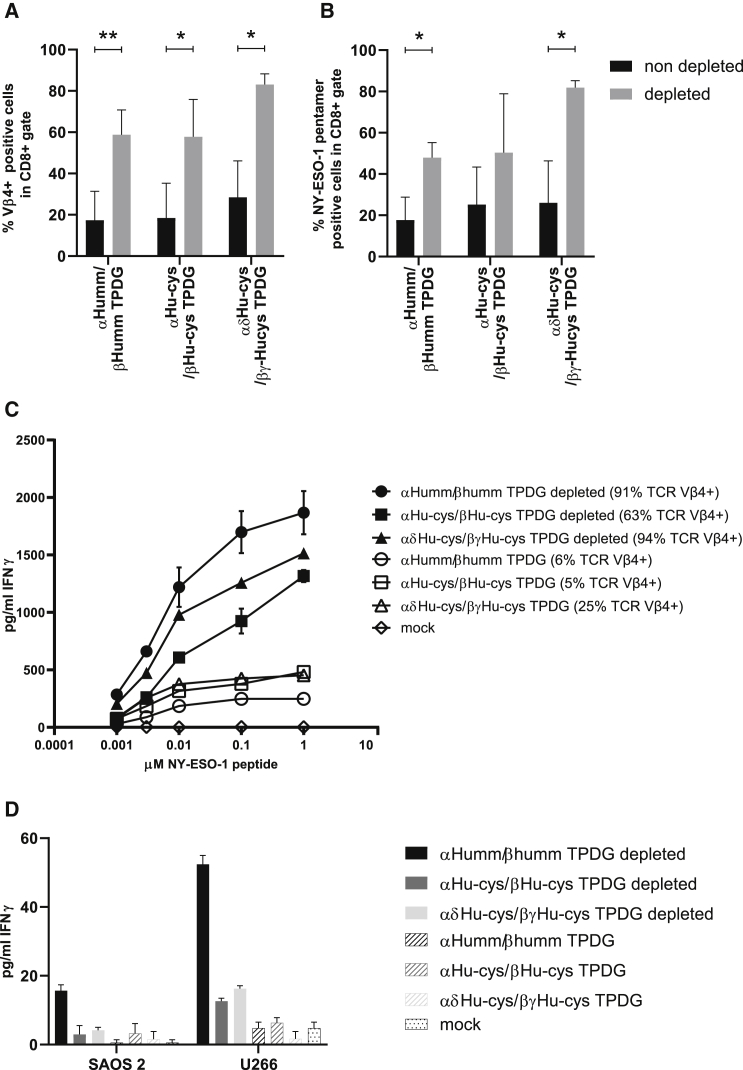
Depletion of non-engineered and poorly engineered T cells within the context of different preferential αβTCR pairing strategies Primary αβT cells were transduced with the three differently modified αβTCRs as indicated in Figure 4A and depleted with the anti-human αβTCR antibody clone BW242/412. (A) Directly before and after depletion, expression of the introduced βTCR was determined by an anti-Vβ4 antibody. (B) Expression of appropriately paired introduced αTCR and βTCR chains was determined by NY-ESO-1 pentamers. (C and D) Functionality of purified or non-purified engineered immune cells was assessed in a stimulation assay after co-incubation with NY-ESO-1_157–165_ peptide-pulsed T2 cells (C) or tumor cell lines with endogenous expression of NY-ESO peptide (D). IFNγ production was measured in the supernatant by ELISA. The data correspond to three (A and B) or two (C and D) independent experiments and are shown as average with standard deviation (A and B) or a representative figure (C and D). Statistical significance (∗p ≤ 0.05, ∗∗p ≤ 0.01) was calculated using a one-tailed paired t test.

Multiple alternative strategies to prevent αβTCR chain mispairing and thereby increase the expression of the introduced tumor specific αβTCR have been reported. For example, adding an additional cysteine residue to introduce a disulfide bridge between the α and β chains has been shown to increase expression and decrease mispairing.^8^ Also, human γδTCRs introduced in human T cells do not pair with endogenous αβTCRs.^35^ Therefore, it was attractive to use γδTCR transmembrane domains for engineering αβT cells in a similar way. We tested whether our enrichment strategy could also be combined with these alternative pairing solutions. First, we constructed an NY-ESO-1-specific TCR with an additional disulfide bridge by the mutation of one specific residue in each chain, i.e., T48C in TCRCα and S57C in TCRCβ.^8^ Second, we constructed an NY-ESO-1-specific TCR with the same additional disulfide bridge, and with a human γδTCR transmembrane domain. These TCRs were compared to the previously used mm TCR strategy (schematic representation, Figure 4A). To later make use of the αβTCR depletion method, we introduced the mutations T110P+D112G in the β chains. We then assessed the expression of the different TCRs in primary T cells by measuring the percentage of Vβ4^+^ and NY-ESO-1_157-165_ HLA∗02:01 pentamer^+^ cells within the CD8^+^ population (Figure 4B). All three conditions resulted in a NY-ESO-1_157-165_ HLA∗02:01 pentamer^+^ CD8^+^ fraction comparable in size to the Vβ4^+^ CD8^+^ fraction, indicating that in all cases the introduced TCR chains are preferentially paired (Figures 4B and S3). A modest, but significant, increase in expression of the introduced TCR was observed when using a combination of a cysteine bridge and the γδ-transmembrane domain when compared to the mm variant (Figure 4C). The increase in expression of Vβ4 was associated with an increase of the single Vβ4^+^ cells to Vβ4/endogenous αβTCR double-positive cells (Figure 4D), indicating that combination of the cysteine bridge and γδ-transmembrane domain was most potent in the downregulation of the endogenous αβTCR. Next, the three different conditions were αβTCR depleted in the same way as before, and the percentage of Vβ4^+^ cells (Figure 5A) and NY-ESO-1_157–165_ HLA∗02:01 pentamer^+^ cells within the CD8^+^ population (Figure 5B) was measured by flow cytometry, showing successful enrichment for transduced cells in all conditions. After depletion, however, we did not see significant differences in %Vβ4 or pentamer^+^ cells between the three tested constructs. In summary, all three described methods were suitable for creating preferential pairing and subsequent purification by our αβTCR depletion method with a slight advantage of combination of the cysteine bridge and γδ-transmembrane domain when assessed by TCR expression.

### Augmented *in vitro* tumor cell recognition by purified engineered T cells

To assess whether purified NY-ESO-1_157–165_ αβTCR-engineered T cells were superior in target cell recognition compared to non-purified cells, we pulsed T2 cells with multiple concentrations of NY-ESO-1_157–165_ peptide. Purified engineered T cells showed a stronger response to the peptide-loaded T2 cells than to the non-purified cells. Furthermore, we observed that interferon (IFN)γ release was associated with positivity for the different introduced TCRs (Figure 5C). Purification also resulted in the improved recognition of endogenously processed and presented peptide in the NY-ESO-1-positive tumor cell lines Saos-2 and U226 when assessed by IFNγ release (Figure 5D). As we observed varying and only minor differences between the three strategies (Figures 4 and 5), and wanted to introduce as few changes as possible in engineered TCRs, the mm approach was used in the next set of experiments to prevent mispairing and increase expression of the introduced TCR as reported.^29^ The placement of these 9 murine aa, not on the surface but rather buried within the TCR, makes it unlikely that they would cause immunogenicity of the mm TCR as suggested by Sommermeyer et al.^29^

### Developing an antibody recognizing the introduced mutated region

**Figure 6:**
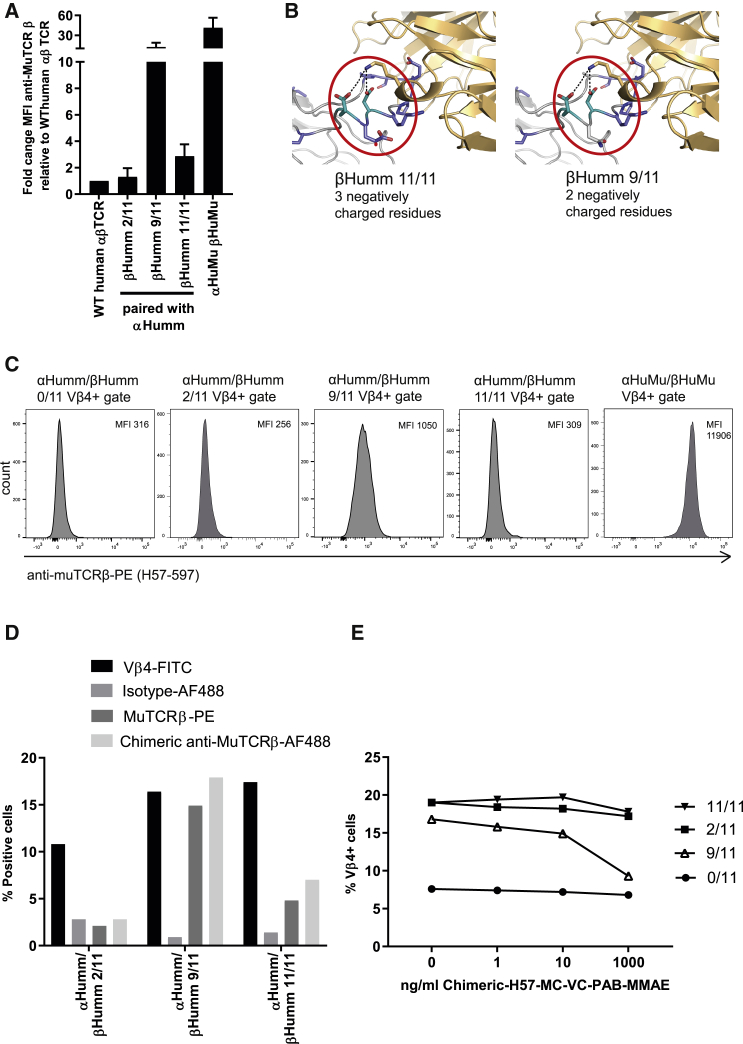
Opportunities for depletion of engineered T cells by using a mutation-specific antibody (A) Jurkat-76 cells were transduced with five different murinized αβTCRs to assess binding of anti-MuTCRβ. Wild-type (WT) αβTCR-transduced Jurkat-76 cells served as a negative control, while Jurkat-76 cells transduced with a TCR containing a complete murine constant domain served as a positive control. (B) The structure of the murinized constant domains (βHumm 11/11 and βHumm 9/11) when binding of H57-597 was modeled on the template of the β chain of the murine N15 T cell receptor (PDB: 1NFD).^49^ (C) Primary αβT cells were transduced with the five different murinized αβTCRs, and co-staining was performed with anti-MuTCR and anti Vβ4 antibodies. Cells were first gated for Vβ4 positivity, and plots of the anti-MuTCR MFI in the Vβ4-positive gate are shown. (D) Primary αβT cells expressing three different murinized αβTCRs were used to assess binding of wild-type and chimeric anti-MuTCRβ. Anti-Vβ4 and anti-human IgG1-AF488 isotype were included as a positive and a negative control, respectively. (E) Jurkat-76 expressing four different murinized αβTCRs were incubated with chimeric H57-MC-VC-PAB-MMAE for 24 h and then stained with an anti-Vβ4 antibody. The data correspond to one experiment (C), two independent experiments (D and E) for which a representative figure is shown, or three independent experiments (A) shown in a bar graph representing average and standard deviation.

The infusion of engineered T cells can potentially be toxic, due to the occurrence of cytokine release syndrome^13^ or the off-target toxicity of the receptor used.^14^ To be able to deplete infused engineered T cells *in vivo* when deemed necessary, we first sought to raise an antibody specific for the T110P+D112G murinized variant of the αβTCR by immunizing three Wistar rats with a human-mouse chimeric peptide. Despite that antibodies were formed against the chimeric peptide (Figure S4A), no antibody binding to surface-expressed αβTCRs could be detected (Figure S4B). Therefore, we assessed whether the commercially available anti-murine TCRβ chain antibody clone H57-597 (from now on referred to as anti-MuTCRβ) was able to bind the murinized αβTCRs on Jurkat-76 cells generated so far. Jurkat-76 cells expressing the T110P+D112G murinized variant of the αβTCR (indicated by βHumm 2/11; i.e., 2 out of the 11 non-homologous aa in the third domain are murinized) were not bound by anti-MuTCRβ; however, Jurkat-76 cells expressing the βHummM3 murinized variant of the αβTCR (indicated by βHumm 11/11; i.e., all 11 non-homologous aa in the third domain are murinized) were bound by anti-MuTCRβ (Figure 6A). To limit the amount of murine aas introduced, we also constructed a variant in which 9 out of 11 non-homologous aa in the third domain are murinized (Figure S4C). Both 11/11 and 9/11 non-homologous murine aa in the β chain of domain 3 were sufficient to reestablish binding of anti-MuTCRβ, but not to the same extent as the HuMu αβTCR (Figure 6A). Surprisingly, 9/11 caused a higher mean fluorescence intensity (MFI) than did 11/11. Structural analyses suggested that this differential binding could be a consequence of the fact that 9/11 contains one less negatively charged residue and therefore results in a more focused electrostatic potential to attract the lysine on CDR1 of anti-MuTCRβ (Figure 6B). To confirm that the anti-MuTCRβ antibody binds to the Vβ4^+^ cells, co-staining was performed with both antibodies on transduced primary T cells. The MFI of anti-MuTCRβ-PE was plotted for the Vβ4^+^ gated cells, which showed that the anti-MuTCRβ antibody bound best to the 9/11 or complete murine constant domain (Figure 6C). As expected, there was no binding to the 2/11 variant but surprisingly also not to the 11/11 variant. This might suggest some interference when both antibodies are used in a co-staining, mainly affecting the suboptimal anti-muTCRβ binding to the 11/11 variant.

Since the clone of anti-MuTCRβ antibody is of Armenian hamster origin and presumably induces severe side effects once administered to humans, comparable to anti-thymocyte globulin,^36^ we aimed to generate a humanized variant of anti-MuTCRβ. We generated chimeric variants of anti-MuTCRβ (H57-597, PDB: 1NFD) by exchanging the hamster immunoglobulin (Ig)G2 constant domain for the human IgG1 constant domain (referred to as chimeric anti-MuTCRβ). We tested binding of this newly constructed antibody in engineered Jurkat-76 cells, which resulted in specific antibody binding to the 9/11 murinized TCRβ chain expressed on Jurkat-76 cells (Figure S5). To determine the capacity of the chimeric anti-MuTCRβ antibody to bind to primary T cells expressing the murinized αβTCRs, we conjugated this antibody and an isotype control to Alexa Fluor 488 (AF488) and determined binding by flow cytometry. The chimeric anti-MuTCRβ antibody was able to bind both 9/11 and 11/11 murinized TCRs and, as shown in Figure 6A, the binding to 9/11 was stronger than to 11/11 (Figure 6D). To assess whether the chimeric variant of anti-MuTCRβ was able to selectively deplete engineered T cells *in vitro*, the antibody was coupled to monomethyl auristatin E (MMAE), a cell cycle inhibitor, using the protease cleavable linker VC-PAB^37^ to create an antibody-drug conjugate (ADC). Jurkat-76 cells transduced with different murinized TCRs were incubated with multiple concentrations of the ADC. The highest concentration of chimeric H57-MC-VC-PAB-MMAE led to a decrease of Vβ4 positivity in the 9/11 condition only (Figure 6E). This specific decrease indicated that the ADC is able to selectively deplete 9/11, and not 11/11, αβTCR-engineered Jurkat-76 *in vitro*, most likely due to the weaker binding of the engineered antibody to the 11/11 αβTCR (Figure 6D). However, depletion was far from complete, indicating that although this binding site is interesting, it is far from being developed for a kill strategy.

## Discussion

The main finding of our study is that replacing only 2 aa within the constant domain of the TCRβ chain allows for the purification of αβTCR-engineered T cells with GMP-ready tools,^38^ without the need for additional complex genetic engineering. The very same region on the TCRβ chain can also serve as a targeting interface for antibodies, which can be used to develop strategies to eliminate engineered immune cells. These new insights provide the molecular basis for developing select-kill strategies for increasing purity and augmenting the safety of αβTCR engineered T cells, with only minor engineering steps.

A sufficient downregulation of the endogenous αβTCR chains by the introduced αβTCR chains is essential for this method to work. Therefore, strategies interfering with endogenous αβTCRs or utilizing knockout of the α or β locus to enhance expression of introduced αβTCRs^39^ will benefit from this strategy. However, engineering T cells via zinc finger nucleases (ZFNs), CRISPR, or transcription activator-like effector nucleases (TALENs)^40^ requires additional engineering steps and therefore is an additional hurdle for GMP-grade production. We accomplished dominance of the introduced receptors by using a previously described method where human residues are replaced by key murine counterparts.^29^ Furthermore, we successfully assessed whether the introduction of an additional disulfide bridge^8^ or the exchange of the human αβTCR transmembrane domain for the human γδTCR counterpart^19^ could also lead to enhanced expression. Thus, we found, in line with our recently published solution for TEGs,^19^ an elegant and minimalistic strategy to purify αβTCR-engineered T cells.

We observed, as reported previously for purification of TEGs,^19^^,^^20^ that αβT cells double positive for endogenous and introduced TCRs are also depleted. This is most likely due to the high affinity of the GMP-grade depletion antibody to the natural βTCR chain. This resulted in a substantial loss of engineered immune cells with residual endogenous αβTCR expression. Although the purified population represented only a small fraction of the initial population, we have shown when using this process for γδTCRs engineered immune cells that the recovery is sufficient to reach therapeutic cell numbers in a full GMP-grade process.^20^ Furthermore, we observed enrichment of NK and γδT cells after depletion, previously reported for γδTCR-engineered immune cells^20^ and transplantation products^41^ as well. Therefore, selection of CD4^+^ and CD8^+^ T cells prior to transduction is recommended when applying our strategy. Selection of CD4^+^ and CD8^+^ T cells is used already successfully during the full grade GMP production process of approved CAR T products.^42^ Overall, our strategy can further improve the current practice for infused engineered products that harbor only between 15% and 55% engineered immune cells,^43^^,^^44^ since the lack of purity of infusion products can become a major clinical obstacle in terms of efficacy^19^ as well as toxicity.^13^^,^^45^

Many tumor-associated antigens targeted by αβTCR gene therapy are not exclusively expressed on tumor cells.^46^ Thus, depending on the type of antigen targeted by the introduced αβTCR, depletion strategies can be useful. This is illustrated by multiple clinical trials, which have led to devastating results caused by off-target or on-target but off-tumor toxicities.^5^^,^^14^ Preclinical strategies to predict off-target toxicities by affinity-enhanced TCRs provide an important tool to minimize these risks.^47^ However, these strategies are not infallible, and therefore an additional safeguard would be extremely valuable when, e.g., targeting novel antigens or antigens that are also partially expressed on healthy tissues. Methods described so far for introducing a safety switch in engineered T cell products rely on the introduction of additional genes for the expression of (truncated) targetable proteins, the introduction of inducible caspase proteins,^48^ or sensitivity to ganciclovir in the case of the widely used HSV-TK suicide gene.^15^ The strategy described herein, using minimal murine aa substitutions, is not only suitable for creating an untouched population of purified T cells, but it also has the potential to develop strategies that will allow an *in vivo* depletion when needed. However, to accomplish this goal, the two identified murine aas that enable αβTCR depletion needed to be expanded with an additional 7 aa to create a chimeric TCRβ chain with a total of 9 murine aa. The major advantage of our strategy, as compared to strategies using, e.g., myc-tags introduced into the TCRα chain,^16^ would be its combined property as a selection and a safeguard system, as well as its use of natural αβTCR domains, which most likely do not affect signaling or impair pairing. However, a major remaining limitation of our approach at this stage is the reduced binding efficacy of our engineered depletion antibody to the murine mutants when compared to the murine wild-type, implying that further engineering of the TCR domain or affinity maturation of the antibody will be needed to enable translation of this strategy into an efficient killing strategy *in vivo*. As binding of the antibody is also partially driven by residues in the Cβ-TCR M1 domain,^49^ additional introduction of several murine aas in this domain could therefore be considered.

In conclusion, the murinization of two specific residues in the TCRβ constant domain allows for the untouched isolation of αβTCR-engineered T cell products, and it can be easily introduced in existing GMP procedures. When a safeguard of engineered immune cells is required, mutating an additional 7 human aa to murine residues in the TCRβ constant domain allows for binding of an antibody, which has the potential to, after further optimization, selectively recognize engineered T cells. However, the second step will require additional engineering of the TCR-antibody interface as well as carefully selecting the appropriate killing mechanism to reach its full potential.

## Materials and methods

### Cells and cell lines

Phoenix-Ampho cells (CRL-3213) were obtained from ATCC and cultured in DMEM (Thermo Fisher Scientific, Breda, the Netherlands) containing 1% penicillin/streptomycin (Pen/Strep) (Invitrogen by Thermo Scientific, Breda, the Netherlands) and 10% fetal calf serum (FCS) (Bodinco, Alkmaar, the Netherlands). The TCRβ^−/−^ Jurma cell line (a derivate of Jurkat J.RT3-T3.5 cells^50^), a kind gift from Erik Hooijberg (VU Medical Center, Amsterdam, the Netherlands), TCRβ^−/−^ Jurkat-76, a kind gift from Miriam Heemskerk (LUMC, Leiden, the Netherlands), and the T2 cell line (ATCC CRL-1992) were cultured in RPMI 1640 + GlutaMAX (Thermo Fisher Scientific, Breda, the Netherlands) containing 1% Pen/Strep and 10% FCS. Cell lines were authenticated by short tandem repeat profiling/karyotyping/isoenzyme analysis. All cells were passaged for a maximum of 2 months, after which new seed stocks were thawed for experimental use. In addition, all cell lines were routinely verified by growth rate, morphology, and/or flow cytometry and tested negative for mycoplasma using a MycoAlert mycoplasma kit (Lonza, Breda, the Netherlands). PBMCs were obtained from Sanquin Blood Bank (Amsterdam, the Netherlands) and isolated by Ficoll-Paque (GE Healthcare, Eindhoven, the Netherlands) from buffy coats. PBMCs were cultured using the previously described rapid expansion protocol (REP)^35^ in RPMI 1640 containing 5% non-typed human serum (Sanquin Blood Bank, Amsterdam, the Netherlands), 1% Pen/Strep (Invitrogen by Thermo Scientific, Breda, the Netherlands), and 50 μM Gibco β-mercaptoethanol (Fisher Scientific, Thermo Fisher Scientific, Breda, the Netherlands) (collectively called HuRPMI).

### Cloning of TCR chains into single retroviral vectors

The minimally murinized Vα16.1 and Vβ4.1 chains from an NY-ESO1_157–165_/HLA∗02-specific TCR, respectively named M2.2.3 and M1.KA,4.1, were generated as previously described.^29^ Additional partially murinized (regions or single residues) TCR chains were ordered from GeneArt (Life Technologies, Thermo Fisher Scientific, Breda, the Netherlands) or constructed via mutagenesis PCR. Cysteine-modified chains were designed as reported previously.^8^ Variants of chimeric αβ/γδ TCRs were composed using the IMGT database (http://www.imgt.org). Sequences were codon optimized and ordered in an industrial resistance, gene-harboring vector or as DNA strings (GeneArt Life Technologies, Thermo Fisher Scientific, Breda, the Netherlands). DNA strings were processed using the TA TOPO cloning kit (Thermo Fisher Scientific, Breda, the Netherlands) and cloned into the pCR2.1-TOPO vector, according to the manufacturer’s protocol. All TCR chains were cloned separately into the retroviral vector pMP71 between the EcoRI and NotI restriction sites using the indicated restriction enzymes and T4 DNA ligase (all from New England Biolabs, Ipswich, MA, USA). Transformation of ligated constructs was performed in JM109 competent *E. coli* (Promega, Leiden, the Netherlands), and subsequent plasmid DNA isolation was conducted using NucleoBond PC500, according to the manufacturer’s protocol (Macherey-Nagel, Düren, Germany).

### Retroviral transduction of primary T cells and T cell lines

Phoenix-Ampho packaging cells were transfected using FuGENE-HD (Promega, Leiden, the Netherlands) with env (pCOLT-GALV), gagpol (pHIT60), and separate pMP71 constructs containing α or β chains from a NY-ESO1_157–165_/HLA-A∗02-specific TCR (isolated from clone ThP2^51^) kindly provided by Wolfgang Uckert,^29^ or containing TCRγ(G115)-T2A-TCRδ(G115)LM1.^19^ PBMCs preactivated with 50 IU/mL interleukin (IL)-2 (Proleukin, Novartis, Arnhem, the Netherlands) and 30 ng/mL anti-CD3 (clone OKT-3, Miltenyi Biotec, Bergisch Gladbach, Germany), CD4/CD8 T cells selected from PBMCs with a REAlease CD4/CD8 (TIL) MicroBead kit (Miltentyi Biotec, Bergisch Gladbach, Germany) preactivated with anti-CD3/CD28 Dynabeads bead to a T cell ratio of 1:5 (Thermo Fisher Scientific, Breda, the Netherlands), and 1.7 × 10^3^ IU/mL MACS GMP recombinant human IL-7 and 1.5 × 10^2^ IU/mL MACS GMP recombinant human IL-15 (Miltenyi Biotec, Bergisch Gladbach, Germany), and Jurma or Jurkat-76 cells were transduced twice within 48 h with viral supernatant in six-well plates (4 × 10^6^ cells/well) in the presence of 50 IU/ml IL-2 (PBMCs only), 1.7 × 10^3^ IU/ml IL-7, 1.5 × 10^2^ IU/ml IL-15, and CD3/CD28 Dynabeads at a 1:5 bead-to-T cell ratio (CD4/CD8-selected T cells only), and 6 μg/mL Polybrene (all) (Sigma-Aldrich, Munich, Germany). After transduction, primary T cells were expanded by the addition of 50 μL/well anti-CD3/CD28 Dynabeads (Thermo Fisher Scientific, Breda, the Netherlands) and 50 IU/ml IL-2 or 1.7 × 10^3^ IU/mL IL-7 or 1.5 × 10^2^ IU/mL IL-15.

### Purification of engineered T cells by MACS depletion of poorly engineered and non-engineered immune cells

Transduced primary T cells were incubated with biotin-labeled anti-human αβTCR antibody (clone BW242/412; Miltentyi Biotec, Bergisch Gladbach, Germany), followed by incubation with an anti-biotin antibody coupled to magnetic beads (anti-biotin MicroBeads; Miltentyi Biotec, Bergisch Gladbach, Germany).^19^ Next, the cell suspension was applied to an LD column in a QuadroMACS separator. αβTCR^+^ T cells were depleted by MACS cell separation according to the manufacturer’s protocol (Miltentyi Biotec, Bergisch Gladbach, Germany).

### *In silico* TCR modeling

The structure of different murinized constant domains was predicted using SWISS-MODEL^52^ on the modeled template of the β chain of the human JKF6 T cell receptor (PDB: 4ZDH). The structure of the murinized constant domains when binding H57-597 was modeled on the template of the β chain of the murine N15 T cell receptor (PDB entry code: 1NFD).^49^ Structure visualizations were performed using the PyMOL molecular graphics system (https://pymol.org/2/).

### Chimeric antibody production and purification

Hamster-human (IgG1) chimeric H57-597 antibody was generated using Lonza expression vectors (pEE14·4-kappaLC, pEE14·4-IgG1).^53^^,^^54^ The antibody was produced by transient transfection of HEK293F cells with the heavy chain coding plasmid, the light chain coding plasmid, and pAdVAntage (accession no. U47294; Promega, Leiden, the Netherlands) using 293fectin transfection reagent (Invitrogen, Thermo Scientific, Breda, the Netherlands) following the manufacturer’s instructions. Antibody-containing supernatant was harvested 4 days after transfection and purified by affinity chromatography using HiTrap protein G HP antibody purification columns (GE Healthcare, Eindhoven, the Netherlands).

### Sequencing

DNA sequences of cloning intermediates and final constructs in pMP71 were verified by barcode sequencing (BaseClear, Leiden, the Netherlands). 75 μg of plasmid DNA and 25 pmol of primer specific for the pCR2.1-TOPO vector or pMP71 vector were premixed in a total of 20 μL and sent to BaseClear for Sanger sequencing.

### Flow cytometry

Cells were stained with Vβ4-fluorescein isothiocyanate (FITC) (TRBV29-1, clone WJF24; Beckman Coulter, Brea, CA, USA), αβTCR-phycoerythrin (PE) (clone BW242/412; Miltentyi Biotec, Bergisch Gladbach, Germany), CD3-Pacific Blue (PB) (clone UCHT1; Becton Dickinson [BD]), CD4-PeCy7 (clone RPA-T4; eBioscience, Thermo Fisher Scientific, Breda, the Netherlands), CD8-allophycocyanin (APC) (clone RPA-T8; BD), CD8-PB (clone SK1; BioLegend, San Diego, CA, USA), or RPE-conjugated NY-ESO-1_157–165_ HLA∗02:01 (SLLMWITQV) pentamer (ProImmune, Oxford, UK). Samples were fixed using 1% paraformaldehyde (PFA) in PBS, measured on a FACSCanto II flow cytometer (BD, Eysins, Switzerland), and analyzed using FACSDiva (BD, Eysins, Switzerland) or FlowJo (BD, Eysins, Switzerland) software.

### ELISA

Effector and target cells (E:T 50,000:50,000) were incubated for 16 h, after which supernatant was harvested. IFNγ ELISA was performed using an ELISA-ready-go! kit (eBioscience, Thermo Fisher Scientific, Breda, the Netherlands) following the manufacturer’s instructions.

### MMAE ADC construction

Chimeric H57-MC-VC-PAB-MMAE was constructed using a kit from CellMosaic (Woburn, MA, USA) following the manufacturer’s instructions.

### Statistical analysis

Statistical analyses were performed using GraphPad Prism 8.3.0 for Windows (GraphPad, La Jolla, CA, USA). Differences between groups was calculated using a one- or two-tailed paired t test (Figures 3 and 5) or a repeated-measures one-way ANOVA (Figure 4). Normal distribution of input data was assumed.

## Supplemental information

Document S1. Figures S1–S5 and Table S1

Document S2. Article plus supplemental information
