# Supplementary material for: Characterization and modulation of anti-αβTCR antibodies and their respective binding sites at the βTCR chain to enrich engineered T cells
Source: Mol Ther Methods Clin Dev. 2021 Jun 24;22:388–400. doi: 10.1016/j.omtm.2021.06.011 (PMC8411211; doi:10.1016/j.omtm.2021.06.011)
Supplement: Document S1. Figures S1–S5 and Table S1 [file mmc1.pdf]

OMTM, Volume *22*

Supplemental information

Characterization and modulation of anti-αβTCR antibodies and their respective binding sites at the βTCR chain to enrich engineered T cells

Guido J.J. Kierkels, Eline van Diest, Patricia Hernández-López, Wouter Scheper, Anja C.M. de Bruin, Elselien Frijlink, Tineke Aarts-Riemens, Sanne F.J. van Dooremalen, Dennis X. Beringer, Rimke Oostvogels, Lovro Kramer, Trudy Straetemans, Wolfgang Uckert, Zsolt Sebestyén, and Jürgen Kuball

Supplemental Figure 1

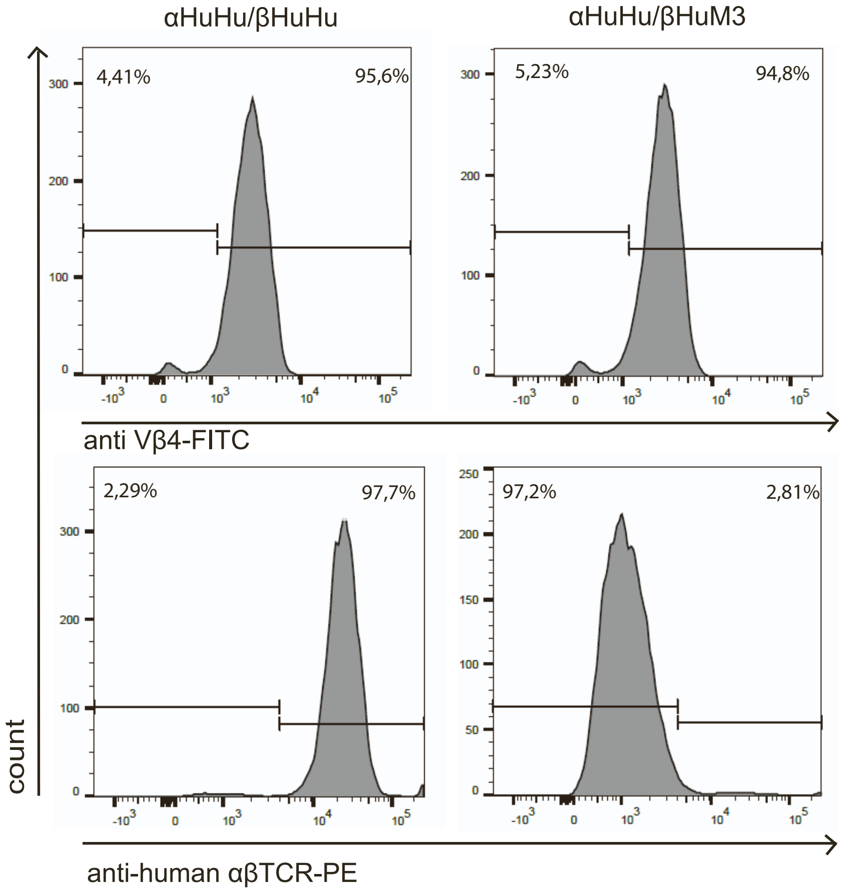

Supplemental Figure 1. αhuhu/βhuM3 murinized TCR is expressed at the cell surface. Jurma cells were transduced with the αhuhu/βhuhu and αhuhu/βhuM3 murinized TCR after which TCR expression was confirmed with an anti-Vβ4 antibody (upper panel) and binding of the anti-human αβTCR antibody was assessed (lower panel), by flow cytometry. The data correspond to 2 independent experiments and a representative figure is shown.

Supplemental Figure 2

TCRα

```
                                1                                       
                                                                          
     1          11         21         31         41         51         61
Hu   IQNPDPAVYQ LRDSKSSDKS VCLFTDFDSQ TNVSQSKDSD VYITDKTVLD MRSMDFKSNS AVAWSNKSDF
Mu   ****E***** *K*PR*O*ST L********* I**PKTME*G TF******** *KA**S***G *I****OTS*

                  2                          3
     71         81         91         101        111        121        131
Hu   ACANAFNNSI IPEDTFFPSP ESSCDVKLVE KSFETDTNLN FQNLSVIGFR ILLLKVAGFN LLMTLRLWSS
Mu   T*QDI*K--- -ETNATY**S DVP**AT*T* ******M*** ******M*L* ********** **********
```

TCRβ

```
                             1                                                 2
     1          11         21         31         41         51         61
Hu   EDLKNVFPPE VAVFEPSEAE ISHTQKATLV CLATGFYPDH VELSWWVNGK EVHSGVSTDP QPLKEQPALN
Mu   ***R**T**K *SL****K** *ANK****** ***R**F*** ********** ********** *AY**---S*

                                        3
     71         81         91         101        111        121        131
Hu   DSRYCLSSRL RVSATFWQNP RNHFRCQVQF YGLSENDEWT QDRAKPVTQI VSAEAWGRAD CGFTSESYQQ
Mu   Y*-******* *******H** ********** H****E*K*P EGSP*****N I********* **I**A**H*

                           4
     141        151        161        171
Hu   GVLSATILYE ILLGKATLYA VLVSALVLMA MVKRKDSRG
Mu   ********** ********** ****G***** ***K*N*--
```

Supplemental Figure 2. Extensive homology between human and murine TCR chains. Sequence alignment of the Human (Hu) and Murine (Mu) TCR α (upper panel) or β (lower panel) constant chains. The three (TCRα) or four (TCRβ) TCR constant regions with clustered Hu-Mu sequence differences are indicated above the alingment.

Supplemental Figure 3

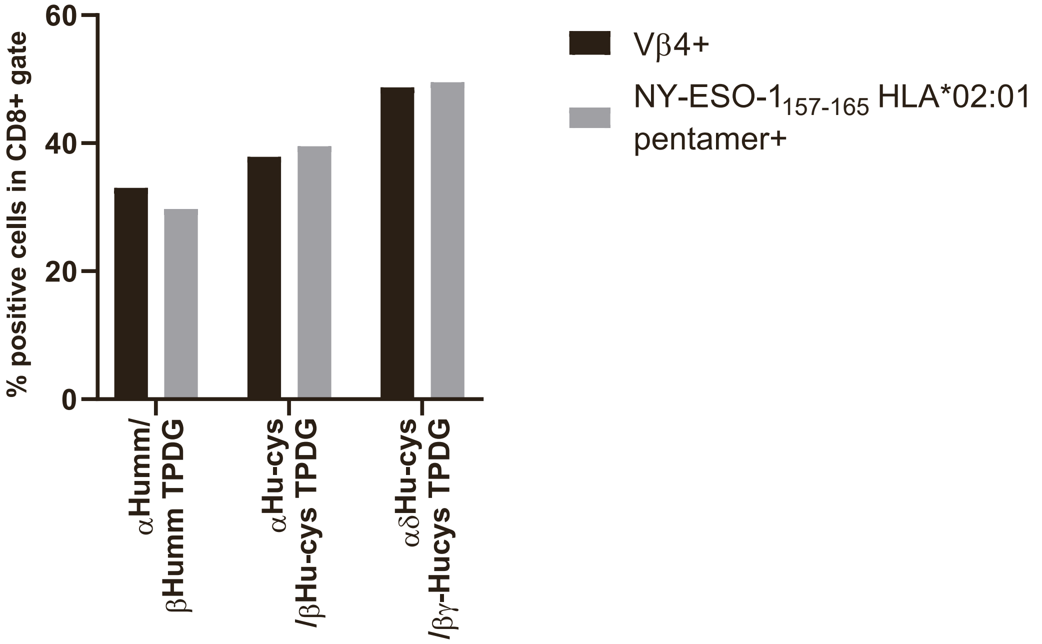

Supplemental Figure 3. Comparable efficacy of different strategies to induce preferential pairing of introduced α and βTCR chains. Primary αβT cells were transduced with the 3 differentially modified αβ TCRs and expression of the introduced βTCR was determined by an anti-Vβ4 antibody. Pairing of the introduced α and βTCR chains was assessed by staining with NY-ESO-1 pentamers. The data correspond to 2 independent experiments and a representative figure is shown.

Supplemental Figure 4

A

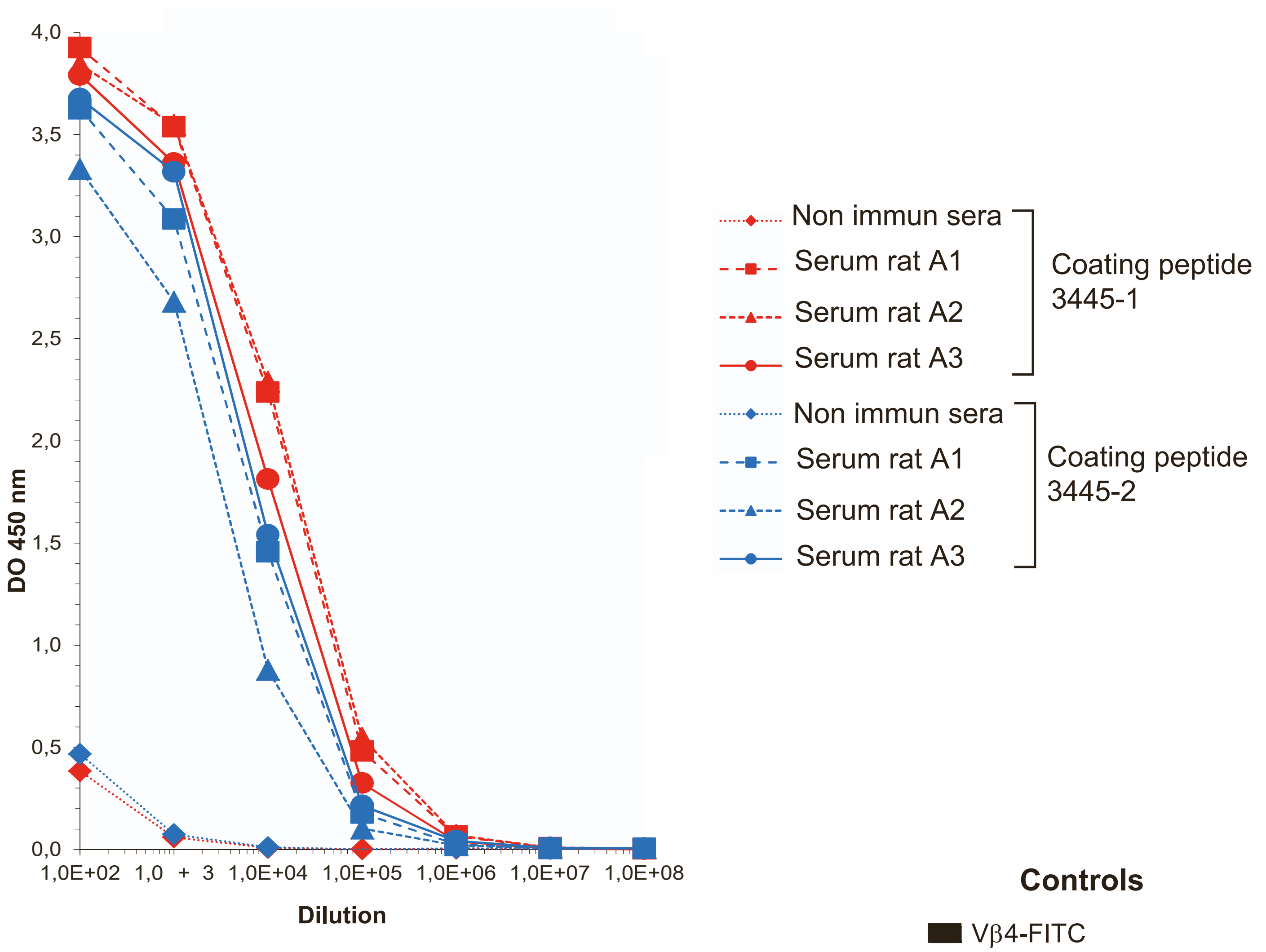

B

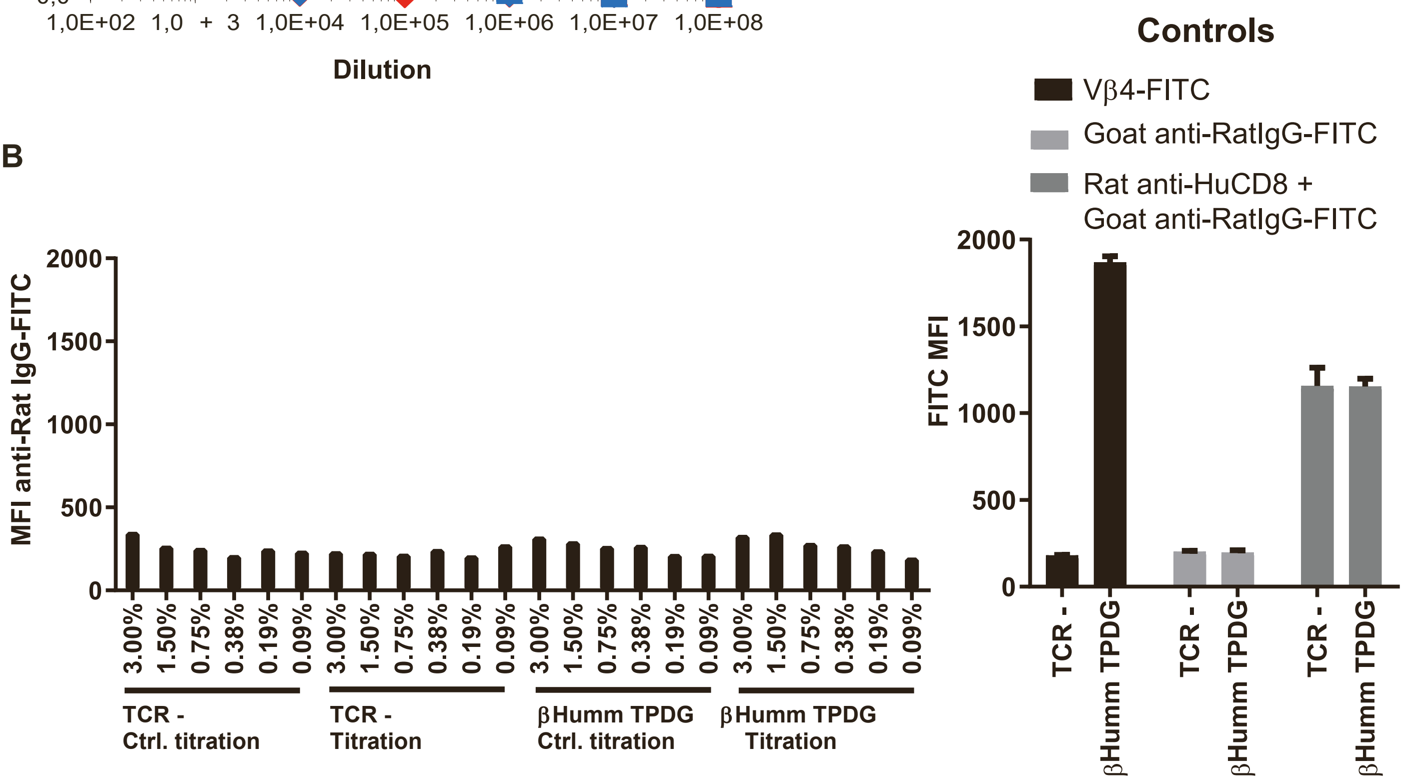

C

```
                                        91          101          111          121
Human                             QNP  RNHFRCQVQF  YGLSENDEWT  QDRAKPVTQI   V
11/11  (Murine)                   H..  ..........  H....E.K.P  EGSP.....N   I
2/11                              ...  ..........  .........P  .G........   .
9/11                              H..  ..........  H......K.P  EGS......N   I
```

Supplemental Figure 4. Attempting to raise an antibody specific for the T110P+D112G murinized variant of the αβTCR by immunizing 3 Wistar rats with a human-mouse chimeric peptide. (A) Determining the presence of peptide-specific antibodies in the serum of the immunized rats. (B) Assessing the ability of the generated antibodies to bind surface-expressed TCRs. aHumm/βHumm TPDG transduced or non-transduced Jurkat-76 cells were incubated with the indicated percentage of rat serum, after which flow cytometry using anti-RatIgG-FITC was performed. In the controls panel, the functionality of this secondary antibody was confirmed by staining the Jurkat-76 cells with rat anti-HuCD8 followed by anti-RatIgG-FITC. Expression of the TCR was confirmed using anti-Vβ4-FITC. (C) Sequence alignment of the human and murine 3rd domain of the TCRβ chain and the constructed 2/11 and 9/11 murinized variants.

Supplemental Figure 5

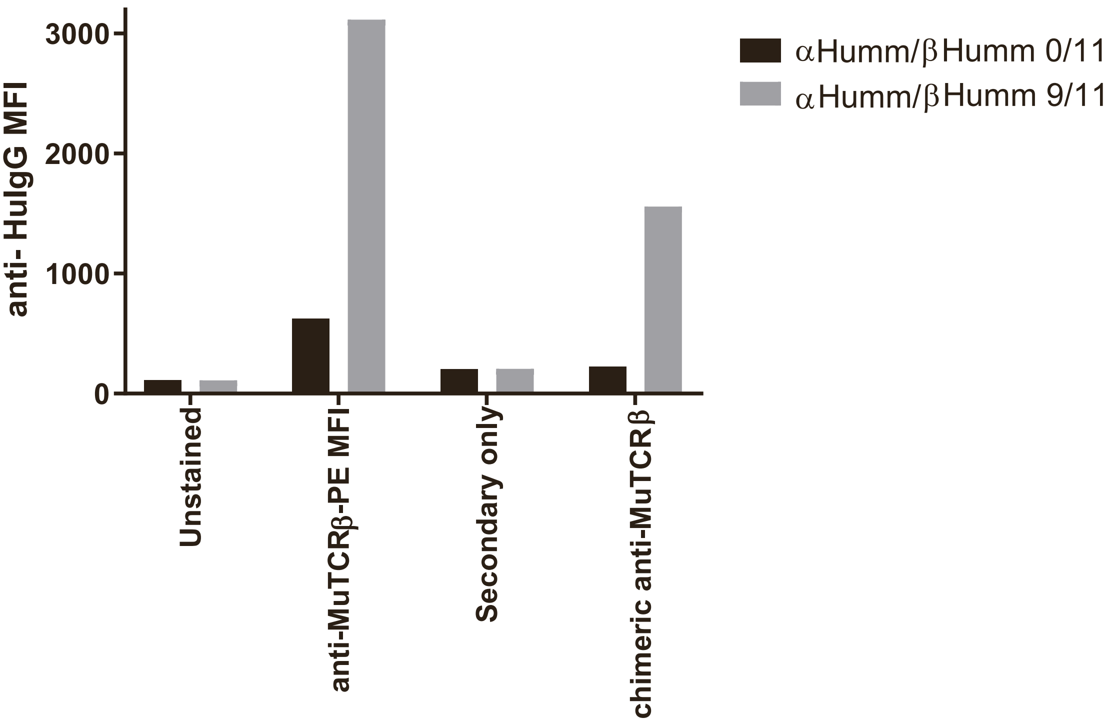

Supplemental Figure 5. Chimeric anti-MuTCRβ antibody binds to primary T cells expressing the murinized TCR containing 9 out of 11 murine residues in the 3rd domain of the β chain. Jurkat-76 cells were transduced with 2 different αβTCRs, containing 0/11 or 9/11 murine residues in the 3rd domain of the β chain, to assess binding of the newly generated chimeric and CDR grafted anti-MuTCRβ antibodies. As negative controls, unstained and secondary antibody only conditions were used. As a positive control, wild-type PE-conjugated anti-MuTCRβ was used The data correspond to 2 independent experiments and a representative figure is shown.

Supplemental Table 1. Differences between the eleven human-mouse non-homologous amino acids in the third domain of the β chain (βM3).

	Change in		
Mutation	Size	Charge	Hydrophobicity
$Q_{88}H$	-	Uncharged to positive charge	Hydrophilic to hydrophobic
$Y_{101}H$	-	Uncharged to positive charge	-
$N_{106}E$	-	Uncharged to negative charge	-
**$E_{108}K$**	**-**	**Negative to positive charge**	**Slightly more hydrophilic**
**$T_{110}P$**	**Less bulky**	Uncharged	**-**
$Q_{111}E$	-	Uncharged to positive charge	-
**$D_{112}G$**	**Less bulky**	**Negative charge to uncharged**	**Less hydrophilic**
$R_{113}S$	Less bulky	Positive charge to uncharged	-
$A_{114}P$	-	Uncharged	Less hydrophobic
$I_{120}N$	-	Uncharged	Hydrophobic to hydrophilic
$V_{121}I$	-	Uncharged	-
